# Food Availability and Animal Space Use Both Determine Cache Density of Eurasian Red Squirrels

**DOI:** 10.1371/journal.pone.0080632

**Published:** 2013-11-12

**Authors:** Ke Rong, Hui Yang, Jianzhang Ma, Cheng Zong, Tijiu Cai

**Affiliations:** 1 College of Wildlife Resource, Northeast Forestry University, Harbin, China; 2 School of forestry, Northeast Forestry University, Harbin, China; University of Pretoria, South Africa

## Abstract

Scatter hoarders are not able to defend their caches. A longer hoarding distance combined with lower cache density can reduce cache losses but increase the costs of hoarding and retrieving. Scatter hoarders arrange their cache density to achieve an optimal balance between hoarding costs and main cache losses. We conducted systematic cache sampling investigations to estimate the effects of food availability on cache patterns of Eurasian red squirrels (*Sciurus vulgaris*). This study was conducted over a five-year period at two sample plots in a Korean pine (*Pinus koraiensis*)-dominated forest with contrasting seed production patterns. During these investigations, the locations of nest trees were treated as indicators of squirrel space use to explore how space use affected cache pattern. The squirrels selectively hoarded heavier pine seeds farther away from seed-bearing trees. The heaviest seeds were placed in caches around nest trees regardless of the nest tree location, and this placement was not in response to decreased food availability. The cache density declined with the hoarding distance. Cache density was lower at sites with lower seed production and during poor seed years. During seed mast years, the cache density around nest trees was higher and invariant. The pine seeds were dispersed over a larger distance when seed availability was lower. Our results suggest that 1) animal space use is an important factor that affects food hoarding distance and associated cache densities, 2) animals employ different hoarding strategies based on food availability, and 3) seed dispersal outside the original stand is stimulated in poor seed years.

## Introduction

Food hoarding is an adaptive strategy for animals to overcome periodic or unpredictable variations in food availability [1], [2], [3]. Hoarders spatially redistribute their food resources in a temporal dimension, thus gaining a foraging advantage during periods of food scarcity [4], [5], [6]. However, storage losses caused by cache competitors may decrease the survival of hoarders and their future reproductive capacity [7], [8]. Therefore, hoarded food must be protected from cache competitors to ensure survival [9]. Larder-hoarding and scatter-hoarding are the two typical types of caching behaviors [2], [3]. Larder hoarders typically store most or all food items in one or a few central sites and can effectively enshroud or defend their storage [2], [3], [10], [11], [12]. In contrast, scatter hoarders commonly place one or a few food items in multiple, separate small sites throughout their home range, and these caches are not specifically defended [3], [8], [13]. Hence, cache pilfering is common, and scatter hoarders are likely to be more susceptible to cache pilferers than larder hoarders [14], [15], [16]. A larger hoarding distance from the food source combined with greater cache spacing can reduce pilfering rates but increases the energy costs associated with hoarding [1], [17], [18]. To maximize net energy gains, the scatter hoarders must carefully space their caches and maintain an optimal cache density [15], [17], [19], [20], [21]. Based on these considerations, two optimal density models have been proposed by Stapanian and Smith (1978) and Clarkson et al. (1986) [1], [15]. Both models suggest that hoarding should begin near a food source, with subsequent hoarding occurring at an increasing distance from the source, and that an optimal cache density should be maintained [1], [15], [17], [22]. The two models mainly differ in the suppositions of the optimal cache density pattern. According to Stapanian and Smith (1978), the cache density around a food source should be constant throughout the hoarding area [1], [22]. Nevertheless, a hoarding site far from the food source implies relatively lower food availability and is threatened by more intensive potential food competition [17]. Lower cache density may be required to reduce cache pilfering. Clarkson et al. (1986) proposed that cache density should decrease with an increasing distance from the food source and that large seeds should be placed in caches farther away from the source to reduce major storage losses [15], [22]. According to Stapanian and Smith (1978), the average hoarding distance should be larger in seed mast years because more seeds can be hoarded to maintain optimal cache densities [1], [23]. This suggestion has been supported by several studies [23], [24]. However, other empirical studies have found that hoarding distances are greater in poor seed years than in seed mast years [17], [25], [26], which supports Clarkson et al. (1986).

The conflicting hoarding strategies are possibly due to several variables that may or may not have been considered in these previous studies. For instance, potential cache competitors, seed types, seed sizes, other food supplies and interactions among these factors likely affect cache density [17], [25]. Furthermore, most previous studies on this topic consisted of manipulated or semi-manipulated experiments in small enclosures that consequentially disregard features of the space use behavior of target animals [24]. Remaining in a particular area or home range is an important trait of animal space use and reflects animals’ physiological and behavioral adaptations to certain environmental characteristics [27], [28]. Thus, animal space use likely influences hoarding sites and, therefore, hoarding distances and cache densities. The distances between caches and food sources are determined not only by food availability but also by the overall home ranges of animals, particularly those in isolated habitats [14], [16], [26]. Therefore, more studies are needed to determine the effect of food availability and animal space use on cache density patterns.

The present study investigated cache density changes in response to animal space use and temporal and spatial variations in food availability in Eurasian red squirrels (*Sciurus vulgaris*) in a mixed broadleaf-conifer Korean pine (*Pinus koraiensis*) forest in northeast China. Based on the optimal density model derived from Clarkson et al. (1986) [15], we predicted that (1) heavier pine seeds would be hoarded farther away from pine seed-bearing trees and (2) cache densities would decrease with hoarding distance and increase with food availability. Although the first prediction has been supported by several studies [17], [19], [24], [25], there are relatively few studies that support the second prediction, and these works present conflicting results [17], [23], [25]. Eurasian red squirrels usually scatter hoard Korean pine seeds on the ground around their nest trees [29]. Because the majority of their nests are situated away from pine seed-bearing trees [30], food availability is lower near the nests. Therefore, we also predicted that (3) the cache density in these sites would be lower than in sites where the nest trees are located near the pine parent stands. Few studies have addressed this prediction, which is logical in conjunction with the second prediction. We conducted systematic cache site investigations to test these three predictions.

## Materials and Methods

### Study site

We investigated the hoarding behavior of Eurasian red squirrels in the Liangshui National Nature Reserve (47°7’15” – 47°14’38”N, 128°48’8” – 128°55’46”E) in the Xiaoxing’an Mountains of Heilongjiang Province in northeastern China between 2007 and 2011. The average elevation of the nature reserve is 400 m with 10–15 degree slopes. The natural vegetation in the area consists of mixed broadleaf-conifer forests dominated by Korean pine trees. Most of the original Korean pine forest was cut down before the establishment of the nature reserve. There are remnant patches of pine parent forest totaling approximately 2,375 ha distributed on ridges and isolated by different types of secondary coniferous forests and secondary broad-leaf forests.

Field investigations were conducted at two separate sample plots (LP and HP), which exhibited different pine densities and different seed production patterns. The area of sample plots was 460 ha for LP and 372 ha for HP. Of these plot areas, original Korean pine forest made up 215.1 ha of LP and 166.4 ha of HP. The pine parent stands were located in the center of each sample plot, and the pine densities of LP and HP were 35 and 54 pine trees per ha, respectively. The plots were more than 1 km apart and were divided by a river, which prevented movements of individual squirrels between plots.

Korean pine seeds are mainly scatter hoarded by squirrels and nutcrackers (*Nucifraga caryocatactes*); both animals make pine seed caches under the ground litter for overwintering during autumn. The caches of the nutcrackers or squirrels are similar and contain 2–3 pine seeds per cache. However, the squirrels’ caches can be identified by their teeth marks on the seeds [31].

We tracked eight squirrels with radio collars to observe their overwintering strategies during 21 weeks from 2007 to 2008 [32]. The home range of the squirrels was 2.46±0.09 ha, and there was no overlap in the home ranges of the individual squirrels. We found that each squirrel used 6±1 nests in its home range. The squirrels used distinct nests during each day and retrieved caches around overnight nest trees. Based on the observations in our study site, the Korean pines were the only overwintering food source for squirrels that gradually made caches from the pine seed-bearing trees extending toward nest trees in autumn [29], [32]. Most nests were outside the pine parent stand, but the distances between the nests and pine seed-bearing trees were no longer than 250 m at the sample plots [30]. These results were confirmed by subsequent radio-tracking observations during the four years (2008 to 2011) following the present study (our unpublished data). We treaded the usage of home ranges and nests as traits of squirrel space use in this study.

### Ethics Statement

The study was conducted with the permission of the Liangshui National Nature Reserve. This research did not involve endangered or protected species. Animals handle and radio-collars use both followed the guidelines established by the American Society of Mammalogists [33]. The study was approved by animal ethics committee of college of wildlife resource, Northeast Forestry University.

### Estimation of pine seed availability

Interannual fluctuations in pine seed production were estimated during the study period. A total of 15 mature pine trees were haphazardly selected as sample trees at the two sample plots every year, respectively. The sample trees were evenly distributed within the Korean pine forest at the sample plots. The pine cones were picked and counted from every sample tree when they matured in October. Pine seeds were removed from the cones, dried, and weighted. We calculated the average cone per tree and average seed mass per ha as indicators of food availability at each sample plot. The interannual fluctuations and differences in seed production between sample plots allowed us to investigate the effect of food availability on cache patterns.

Control seed samples were used to investigate the squirrels’ hoarding selectivity based on seed size. From each of the 15 sample trees, we haphazardly selected five cones and uniformly picked 10 seeds from each cone so that a total of 750 seeds were selected as control seed samples at each sample plot every year. The dry mass of the seeds was weighted.

### Investigation design

It was scarcely possible to follow and directly observe the squirrels’ hoarding behavior due to the dense undergrowth in our study site. Therefore, we conducted indirect measures of hoarding locations to examine our predictions. Fixed distance sampling investigations and nest sampling investigations were conducted to examine predictions 1 and 2. The nest sampling investigations were also used to examine prediction 3.

During the fixed distance sampling investigations, we set 30 transects radially extending outward from the Korean pine parent stands in each sample plot. The minimum distance between the transects was greater than 200 m, and samples were collected in a 10 m by 10 m square every 30 m in each transect. We used GPS receivers and digital maps to ensure that every sample was situated at a fixed distance from the nearest parent pine stand. The samples were divided into the following eight categories according to the sample’s distance from the nearest pine parent stands: under the parent stand (0 m) and 30 m, 60 m, 90 m, 120 m, 150 m, 180 m and 210 m from the parent stand.

During the nest sampling investigations, squirrel nest trees were located. We tracked 8-15 squirrels with radio collars to find their nest trees every year and conducted transect investigations to systematically search for squirrel nests. Parallel transects were unsystematically set across the sample plots. The minimum distance between transects was greater than 200 m. The nests were confirmed to be in use based on subsequent observations. Based on our unpublished data, the squirrels usually made the highest cache density approximately 20–30 m from their nest trees. Therefore, a cache investigation sample was collected from a 10 m by 10 m square set 20 m away from every nest tree toward the nearest pine parent stands. To guarantee independence of each sample, we selected the nest trees that were 400 m away from each other and were used by different squirrels. Because the nests were interspersed among sample plots and not located at specific distances to pine parent stands, we divided the nest samples into the following four categories according to distance from the nearest pine parent stands: in a gap of pine parent stands (GP), under parent pine trees (UP), 0–100 m outside of pine parent stands (OH) and 100–200 m outside of pine parent stands (TH). The nest samples in pine parent stands were classified as being within a gap in the parent stand (GP) when the distance between the center of the sample and the nearest pine trees was greater than 20 m. The other nest samples in pine parent stands were treated as being situated under the parent pine trees (UP) when the distance was smaller than 20 m. Twenty samples from each category were collected at each sample plot every year.

The samples were not collected at the same locations every year during either of the two above-mentioned investigations. We treated the sample distances from the nearest pine parent stands as squirrel hoarding distances.

Based on continuous observations of squirrel activities, we conducted all field investigations in the middle of October, which is when the squirrels completed food hoarding in our study site. We recovered ground cover and comprehensively searched the squirrels’ caches. We counted the number of caches in every sample and used that number as an indicator of the squirrel cache density to examine predictions 2 and 3. All of the pine seeds in the caches were collected to measure the dry seed mass with seed coat using an electric balance with a 0.0001 g precision. The average seed mass in a sample was used to examine prediction 1. Our study design allowed us to simultaneously examine the effect of food availability on hoarding patterns between different years and different areas showing contrasting seed production patterns.

### Statistical analyses

The data of cones per tree and seed production could respectively reflect the differences of food availability at individual level and population level. Since the data of cones per tree were count data, we specified a generalized linear model with a Poisson distribution and an identity link function to explore the differences in cone production among years and between sample plots [34]. The response variable was cones per tree, and the explanatory factors were year, sample plot and interaction of year and sample plot.

Meanwhile, the differences between the sample plots in seed production among years were investigated. The data of seed production for each sample plot during the five-year study period met the normality assumption (Shapiro-Wilk W Test, all *P*_ > W_ values were > 0.05), but the variances of the data were heterogeneous that could not be made homogeneous through any transformations. Therefore, we used a generalized linear model with a normal distribution and an identity link function to model seed production as a function of year, sample plot and interaction between year and sample plot [34]. Based on the results of these analyses, we used the variables of year and sample plot as dummy variables for seed availability in the subsequent data analyses.

We generated liner models to detect how food availability and hoarding distance affected the hoarded patterns of seed masses. The seed masses were employed as response variables, while explanatory factors included year, hoarding distance, sample plot and two-way interactions of these variables. The seed mass data from the fixed distance sampling investigations met the normality assumption (Shapiro-Wilk W Test, *W* = 0.99, *P* = 0.48), and we used a square root transformation of the seed mass data from the nest sampling investigations to meet the normality assumption (Shapiro-Wilk W Test, *W* = 0.99, *P* = 0.29). The best models were selected based on minimum AICc values [34], [35]. Based on the selected final model estimations, we conducted several one-way ANOVAs with Tukey-Kramer HSD multi-comparisons to determine how hoarding distance and food availability affected hoarded seed mass. The results from these analyses were used to address prediction 1.

In order to study the squirrels’ hoarding selectivity based on seed size, we conducted a three-way ANOVA to compare differences of seed mass between hoarded pine seeds and the control seeds which we collected from sample trees. Two additional factors were included in the analysis to control for year and sample plot effects.

Because the cache number data in the samples were count data showing over dispersion (the SD was close to the mean), we conducted generalized linear models with a Poisson distribution and an identity link function to explore the effects of food availability and hoarding distance on cache density [34]. The responses variable was cache density, while explanatory factors included year, hoarding distance, sample plot and two-way interactions of these variables. The best models were selected based on minimum AICc values [34], [35]. Based on the selected final model estimations, we conducted several one-way ANOVAs with Tukey-Kramer HSD multi-comparisons to explore how hoarding distance and food availability affected the patterns of cache densities at different sample plots in different years. We excluded the samples without caches (Table 1 and 2) from these analyses.

**Table 1:** Size of the samples in which at least one cache was discovered in the fixed-distance sampling investigations during a 5-year period.

Sample category		Year				
		2007	2008	2009	2010	2011
Sample plot HP	0 m	30	30	30	30	30
	30 m	30	30	30	30	30
	60 m	28	28	30	30	28
	90 m	26	18	30	30	16
	120 m	12	7	25	30	5
	150 m	15	0	21	13	0
	180 m	20	0	23	11	0
	210 m	20	0	25	0	0
Sample plot LP	0 m	30	30	30	30	30
	30 m	30	29	30	30	29
	60 m	28	28	29	30	30
	90 m	29	26	30	30	30
	120 m	25	5	28	27	7
	150 m	15	2	21	13	2
	180 m	17	0	26	26	0
	210 m	20	0	25	11	0

**Table 2:** Size of the samples in which at least one cache was discovered in the nest-sampling investigations during a 5-year period.

Sample category		Year				
		2007	2008	2009	2010	2011
Sample plot HP	GA	20	20	20	20	20
	UP	20	20	20	20	20
	OH	20	13	20	20	2
	TH	20	0	20	20	0
Sample plot LP	GA	20	20	20	20	20
	UP	20	20	20	20	20
	OH	20	15	20	20	6
	TH	20	0	20	20	0

We used the JMP 9.0.2 software package (SAS Institute Inc. 2010) to perform all analyses [36]. All tests of significance were two-tailed, and the significance level was set at 0.05. All averages are presented with their corresponding standard errors (±SE).

## Results

### Pine seed availability

**Figure 1:**
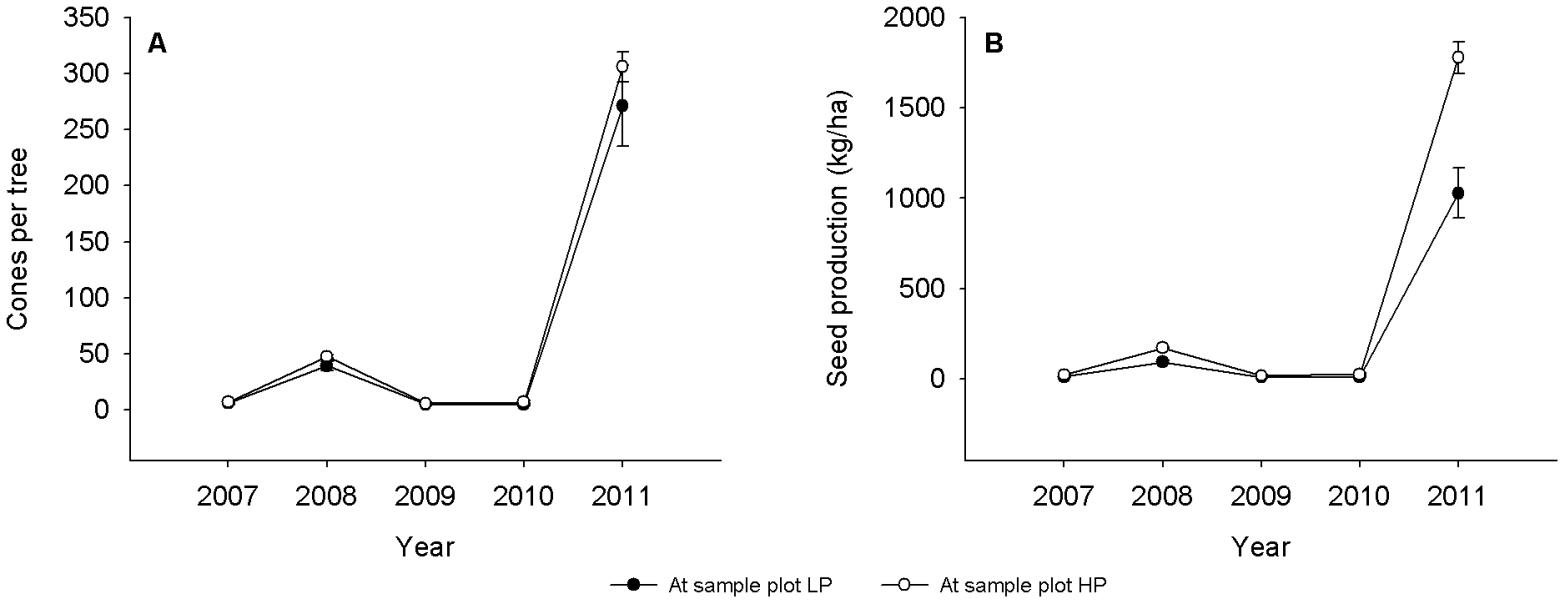
Interannual fluctuations in pine cones per tree and pine seed production at two sample plots in the Liangshui Nature Reserve, Heilongjiang Province, China, over a 5-year period. Results of the GLM model test: figure A, whole model test, Pearson *χ* ^2^ = 1530.74, *df* = 140, *P*<0.0001, overdispersion  =  10.93; year effect, *χ* ^2^ = 1891.79, *df* = 4, *P*<0.0001; effect of sample plot, *χ* ^2^ = 4.44, *df* = 1, *P* = 0.035; interaction effect of year with sample plot, *χ* ^2^ = 3.50, *df* = 4, *P* = 0.48; figure B, whole model test, Pearson *χ* ^2^ = 5.66e+12, *df* = 140, *P*<0.0001; year effect, *χ* ^2^ = 327.37, *df* = 4, *P*<0.0001; effect of sample plot, *χ* ^2^ = 27.04, *df* = 1, *P*<0.0001; interaction effect of year with sample plot, *χ* ^2^ = 66.61, *df* = 4, *P*<0.0001.

There were extreme interannual fluctuations in the availability of Korean pine seeds throughout our study site based on data of both cones per tree and seed production per hectare (FIG. 1). 2011 was a mast year, 2008 was a common year, and the other three years of the study were poor seed years (FIG. 1). The seed availability at sample plot HP was significantly higher than at sample plot LP during every year (FIG. 1).

**Figure 2:**
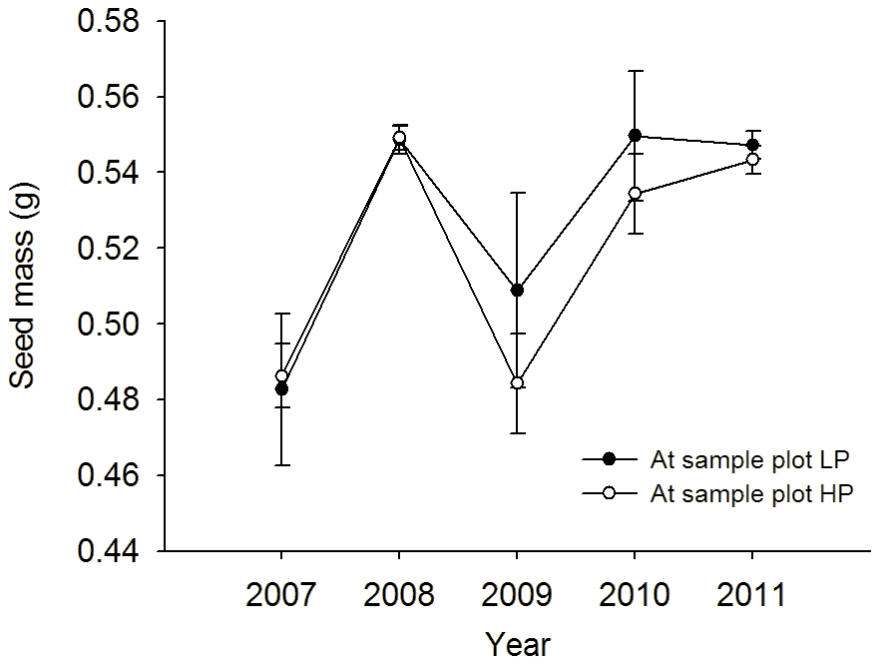
Seed mass of Korean pines collected at two sample plots in Liangshui Nature Reserve, Heilongjiang Province, China, over a 5-year period.

The masses of seeds produced in 2008, 2010 and 2011 were heavier than produced in 2007 and 2009 (0.5454±0.0034 g compared to 0.4904±0.0089 g; ANOVA, *F*_4,145_ = 10.72, *P*<0.0001, FIG. 2). However, there were no significant differences of masses between the seeds produced at the two sample plots (ANOVA, *F*_1,148_ = 0.73, *P* = 0.39, FIG. 2).

### Patterns of seed mass in caches

**Figure 3:**
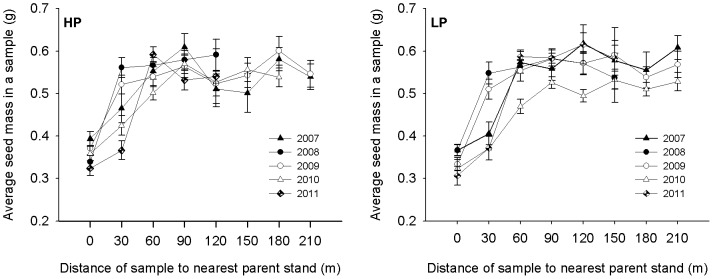
Seed mass of Korean pine seeds hoarded by the Eurasian red squirrel in relation to the hoarding distance in Liangshui Nature Reserve, Heilongjiang Province, China, over a 5-year period. The samples were collected from 10× 10 m squares. Some samples had no caches in (see Table 1) and were excluded from calculations of the average seed mass per sample. The abbreviations HP and LP represent two different sample plots.

We observed an obvious pattern in seed masses from the fixed distance sampling investigations (FIG. 3). The factors of year, hoarding distance and interaction between those two variables were included in the final explanatory model based on minimum AICc values (Table 3). An effect of hoarding distance was detected in the model (Table 3). Under the model estimations, the samples could be divided into three groups according to average seed mass of samples (ANOVA, *F*_7, 1641_ = 73.49, *P*<0.0001). The lightest seeds (0.3484±0.0055 g) were hoarded in group 1 and consisted of 0 m samples; the heavier seeds (0.4545±0.009 g) were hoarded in group 2 and consisted of 30 m samples and the heaviest seeds (0.5400±0.0042 g) were hoarded in group 3 and consisted of 60 m, 90 m, 120 m, 150 m, 180 m and 210 m samples. The seed masses were heaver as hoarding distance extended from 0 m to 60 m, which supports prediction 1 that heavier pine seeds would be hoarded farther away from pine seed-bearing trees. There were no seed mass differences among the samples of group 3 (ANOVA, *F*_5, 1045_ = 1.83, *P* = 0.10). However, a year effect was detected in the model (Table 3). The mass of seeds hoarded in 2008, 2010 and 2011 was significantly lighter than in 2007 and 2009 (0.4589±0.0050 g compared to 0.5217±0.0054 g; ANOVA, *F*_4,1644_ = 19.98, *P*<0.0001). These results suggested that squirrels hoarded heavier seeds when food availability was lower (see FIG 1).

**Table 3:** Models tested for detecting factors influencing seed mass in caches based on fixed-distance sampling investigations.

Linear model	Adjusted *R* ^2^	Analysis of model	AICc
Mass ∼ Year + SDP + SP + ε	0.25	*F*_12,1636_ = 47.76 *P*<0.0001	–1985.37
Mass ∼ Year + SDP + ε	0.25	*F*_11,1637_ = 52.05 *P*<0.0001	–1986.78
Mass ∼ Year + SDP + Year*SDP + ε	0.32	*F*_35,1613_ = 23.60 *P*<0.0001	–2124.54

Abbreviations: SDP  =  sample distances to nearest Korean pine stand, SP  =  sample plot, ε  =  residual.

The last model was selected final explanatory model according to minimum AICc values. Statistical results: ANOVA, Year effect, *F*_4,1613_ = 12.13, *P*<0.0001; Effect of hoarding distance, *F*_7,1613_ = 88.44, *P*<0.0001; Effect of interaction of year and hoarding distance, *F*_24,1613_ = 8.08, *P*<0.0001.

**Figure 4:**
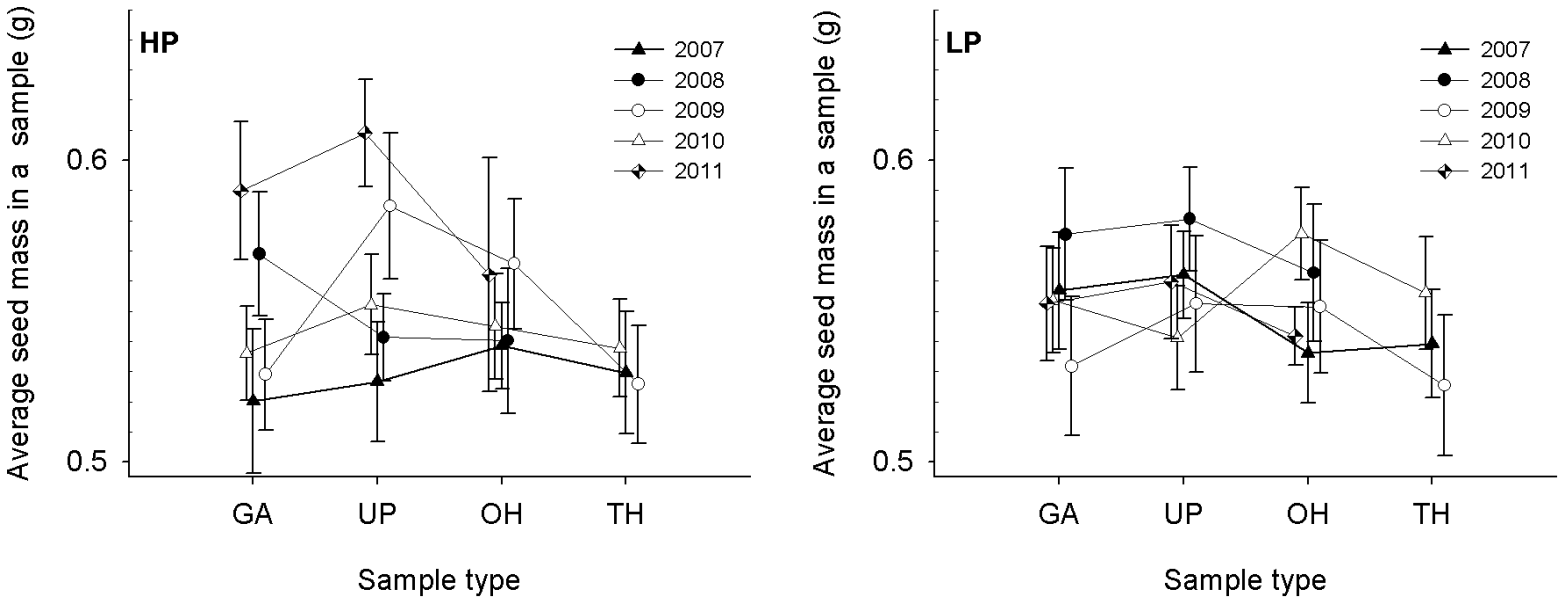
Seed mass of Korean pine seeds hoarded by the Eurasian red squirrel around their nest trees in relation to hoarding distance in Liangshui Nature Reserve, Heilongjiang Province, China, over a 5-year period. The samples were collected in 10× 10 m squares. Some samples had no caches in (see Table 2) and were excluded from calculations of the average seed mass in a sample. The abbreviations HP and LP represent two different sample plots; GA, samples within a gap in a parent pine stand; UP, samples under parent pine trees; OH, samples 0–100 m outside the parent pine stand; TH, samples 100–200 m outside the parent pine stand.

No obvious seed mass pattern was detected from the nest sampling investigations (FIG. 4). The final explanatory model included the variables year, sample plot and interaction of these two variables as explanatory factors. The variable of hoarding distance was not included in the model (Table 4), and the effect of hoarding distance was not detected by one-way ANOVA also (ANOVA, *F*_3,672_ = 2.19, *P* = 0.09). This result, which did not support prediction 1, revealed that the seed masses in the samples around nest trees were not affected by hoarding distance. In the final model, the effects of year and the interaction between year and sample plot were significant, but there was no sample plot effect (Table 4). According to this model, only seed masses hoarded in 2011 were significantly heavier than other years at sample plot HP (0.5978±0.0132 g compared to 0.5428±0.0050 g; ANOVA, *F*_4,330_ = 4.67, *P* = 0.001), and there was no year effect at sample plot LP (ANOVA, *F*_4,336_ = 1.38, *P* = 0.24).

**Table 4:** Models tested for detecting factors influencing seed mass in caches based on nest-sampling investigations.

Linear model	Adjusted *R* ^2^	Analysis of model	AICc
Sqrt(Mass) ∼ Year + ε	0.01	*F*_4,671_ = 3.32, *P* = 0.010	–1932.53
Sqrt(Mass) ∼ SDP + ε	0.01	*F*_ 3.672_ = 2.25, *P* = 0.08	–1928.07
Sqrt(Mass) ∼ SP + ε	0.001	*F*_ 1,674_ = 0.31, *P* = 0.58	–1925.68
Sqrt(Mass) ∼ Year + SP + ε	0.01	*F*_ 5,670_ = 2.71, *P* = 0.020	–1930.76
Sqrt(Mass) ∼ Year + SDP + ε	0.01	*F*_ 7,668_ = 2.37, *P* = 0.022	–1929.69
Sqrt(Mass) ∼ Year + SDP + SP + ε	0.01	*F*_ 8,667_ = 2.10, *P* = 0.034	–1927.89
Sqrt(Mass) ∼ Year + Site + Year*SP + ε	0.02	*F*_ 9,666_ = 2.76, *P* = 0.034	–1933.72

Abbreviations: SDP  =  sample distances to nearest Korean pine stand, SP  =  sample plot, ε was the residual.

The last model was selected as the final explanatory model according to minimum AICc. Statistical tests of the model: ANOVA, Year effect, *F*_4,666_ = 3.47, *P* = 0.008; Effect of sample plot, *F*_1,666_ = 0.004, *P* = 0.95; Effect of interaction of year and sample plot, *F*_4,666_ = 2.78, *P* = 0.03.

The nest sampling investigations revealed that the seeds hoarded by squirrels were heavier than control seeds, while the differences were contrary in the fixed sampling investigations (Table 5). Even the heaviest seeds that were collected from the group of 60 m, 90 m, 120 m, 150 m, 180 m and 210 m samples in the fixed sampling investigations were lighter than the seeds collected from the nest sampling investigations (0.54000±0.0042 g compared to 0.5515±0.0031 g; ANOVA, *F*_1,1725_ = 3.90, *P* = 0.048). These results suggested that squirrels hoarded heavier seeds around their nest trees.

**Table 5:** Comparisons of seed mass between seeds hoarded by squirrels and control seeds collected from sample trees (g)

Sample source	Hoarded seed mass	Control seed mass	Effect tests*
Fixed-distance sampling	0.4897±0.0018	0.5234±0.0018	*F*_1,1792_ = 10.34, *P*<0.001
Nest sampling	0.5515±0.0031	0.5234±0.0018	*F*_1,819_ = 18.26, *P*<0.0001

* Three-way ANOVA with the variables of year and sample plot as two additional factors.

### Patterns of cache density in samples

**Figure 5:**
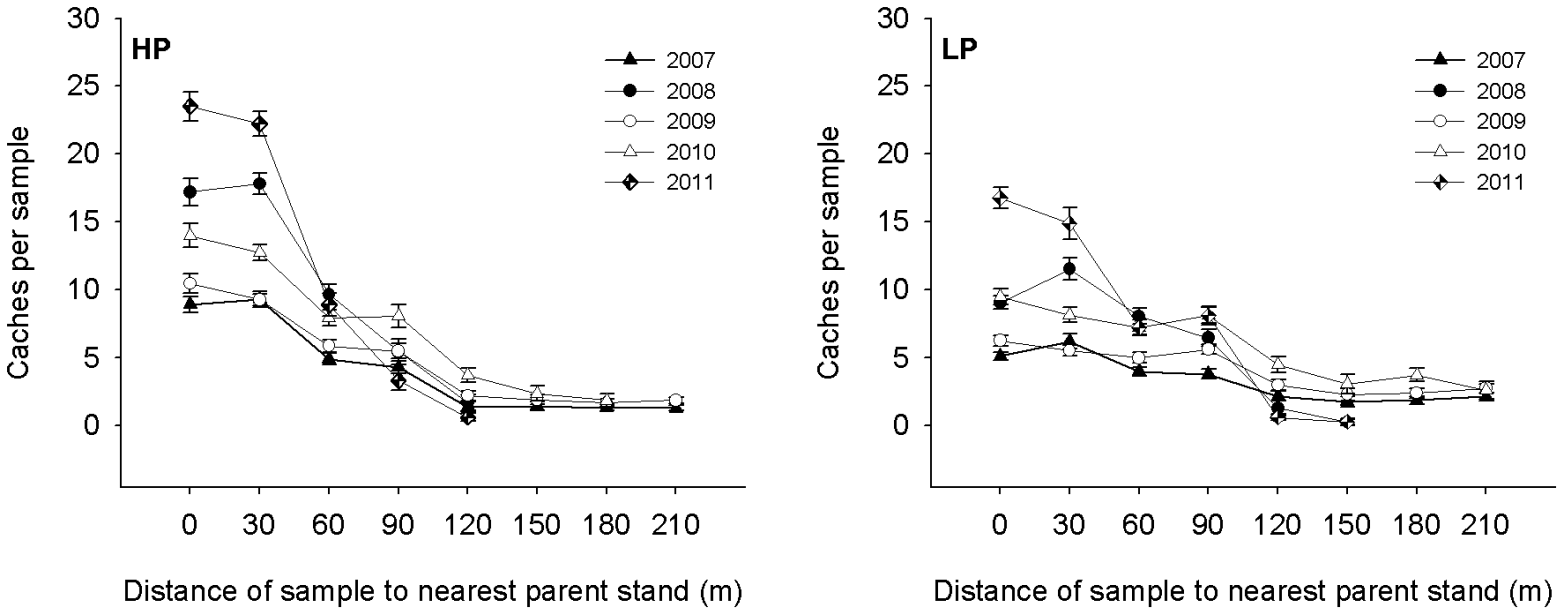
Cache density of Korean pine seeds hoarded by the Eurasian red squirrel (*Sciurus vulgaris*) in relation to the hoarding distance in Liangshui Nature Reserve, Heilongjiang Province, China, over a 5-year period. The samples were collected in 10×10 m squares. The abbreviations HP and LP represent two different sample plots.

The cache density decreased sharply with hoarding distance in the fixed distance sampling investigations (FIG. 5). Based on the model selected with minimum AICc values, the factors of hoarding distance, sample plot and year were included in final explanatory model (Table 6). Under the model estimations, the highest cache densities were detected in the 0 m and 30 m samples; the lower cache densities were detected in the 60 m and 90 m samples and the lowest cache densities were in the 120 m, 150 m, 180 m and 210 m samples (ANOVA, *F*_7,1641_ = 139.26, *P*<0.0001; FIG 5). The cache densities decreased with an increase in hoarding distance, which supported prediction 2. The cache densities at sample plot HP were significantly higher than at sample plot LP (FIG. 5, Table 6), and there were significant year effects (FIG. 5, Table 6). The cache density in different years could be divided into four categories corresponding to 2011, 2008, 2010 and 2009 + 2007 and decreased in that order (ANOVA, *F*_4,1644_ = 196.25, *P*<0.0001). This result also supported prediction 2. Furthermore, the caches were discovered farther away from the pine parent stands in poor seed years (Table 1). Considering interannual fluctuations of seed availability at the two sample plots (FIG. 1), these results showed that the squirrels hoarded farther away from pine parent stands and made lower cache densities when food availability was low.

**Table 6:** Model tested for detecting factors influencing cache densities based on fixed distance sampling investigations.

GLM model	*χ* ^2^	*df*	*P*	Overdispersion	AICc
NC ∼ Year + SDP + SP + ε	2567.04	1639	<0.0001	1.57	5494.70
NC ∼ Year + SDP + SP + Year*SP + ε	2422.73	1632	<0.0001	1.48	5735.26
NC ∼ Year + SDP + SP + Year*SDP + ε	2153.29	1612	<0.0001	1.34	6163.93
NC ∼ Year + SDP + SP + SP*SDP + ε	2222.09	1629	<0.0001	1.36	6087.88

Abbreviations: NC  =  cache density, SDP  =  sample distance to nearest Korean pine stand, SP  =  sample plot, ε  =  residual.

The first model was selected as final explanatory model according to minimum AICc values. Statistical tests of the model: Year effect, *df* = 4, *χ* ^2^ = 612.31, *P*<0.0001; Effect of sample plot, *df* = 1, *χ* ^2^ = 27.22, *P*<0.0001; Effect of hoarding distance, *df* = 3, *χ* ^2^  = 870.54, *P*<0.0001.

**Figure 6:**
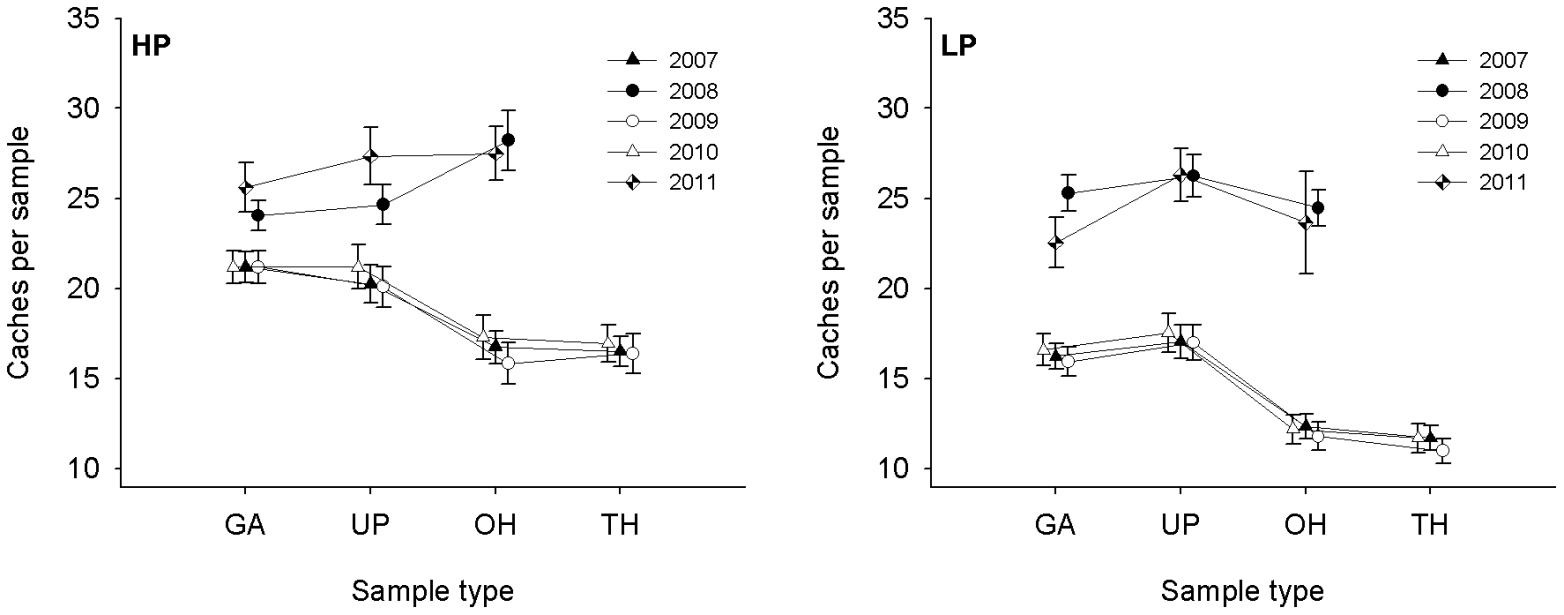
Cache density of Korean pine seeds hoarded by the Eurasian red squirrels (*Sciurus vulgaris*) around nest trees in relation to the hoarding distance in Liangshui Nature Reserve, Heilongjiang Province, China, over a 5-year period. The samples were collected in 10×10 m squares. The abbreviations HP and LP represent two different sample plots; GA, samples within a gap in a parent pine stand; UP, samples under parent pine trees; OH, samples 0–100 m outside the parent pine stand; TH, samples 100–200 m outside the parent pine stand.

A distinct but inconsistent cache density pattern was observed during the nest sampling investigations (FIG. 6). The factors of hoarding distance, sample plot and year were included in the final explanatory model chosen by minimum AICc values (Table 7). High cache densities were detected in the samples at gaps of pine parent stands and under parent pine trees, relatively lower cache densities were detected in the samples 0–100 m outside the pine parent stands and the lowest cache densities were in the samples 100–200 m outside the pine parent stands (ANOVA, *F*_3,672_ = 54.12, *P*<0.0001; FIG. 6). These results support predictions 2 and 3. A sample plot effect and year effect were also detected (FIG. 6, Table 7). The cache densities at sample plot HP were higher than at sample plot LP. The cache density in 2008 and 2011 was higher than in the other years (ANOVA, *F*_4,671_ = 95.64, *P*<0.0001; FIG. 6). These results support prediction 2. To further explore hoarding distance effects in different years, we divided the data into two groups based on years: one group included data collected in 2008 and 2011, and the other group included data collected in 2007, 2009 and 2010 (see FIG. 6). The hoarding distance effects in the 2008 and 2011 group were insignificant (ANOVA, *F*_2,193_ = 2.13, *P* = 0.12), but no caches were found in the samples at 100–200 m outside the pine parent stands (Table 2), indicating that hoarding distances did not affect cache densities in 2008 and 2011. This result did not support predictions 2 and 3. In contrast, there was a clear hoarding distance effect in the group of 2007, 2009 and 2010; the cache densities in the sample at gaps of pine parent stands and under parent pine trees were significantly higher than in the sample 0–100 m and 100–200 outside of the pine parent stands (ANOVA, *F*_3,476_ = 38.24, *P*<0.0001). These results verified predictions 2 and 3 and indicated that cache patterns were based on changes in food availability.

**Table 7:** Model tested for detecting factors influencing cache densities based on nest sampling investigations.

GLM model	*χ* ^2^	*df*	*P*	Overdispersion	AICc
NC ∼ Year + SDP + SP + ε	772.94	667	0.003	1.16	3453.92
NC ∼ Year + SDP + SP + Year*SP + ε	750.27	663	0.010	1.13	3524.22
NC ∼ Year + SDP + SP + Year*SDP + ε	743.40	657	0.011	1.14	3519.34
NC ∼ Year + SDP + SP + SP*SDP + ε	761.63	664	0.005	1.15	3486.06

Abbreviations: NC  =  cache density, SDP  =  sample distance to nearest Korean pine stand, SP  =  sample plot, ε  =  residual.

The first model was selected as the final explanatory model according to minimum AICc values. Statistical tests of the model: Year effect, *df* = 4, *χ* ^2^ = 327.15, *P*<0.0001; Effect of sample plot, *df* = 1, *χ* ^2^ = 114.68, *P*<0.0001; Effect of hoarding distance, *df* = 3, *χ* ^2^  = 118.04, *P*<0.0001.

## Discussion

In place of tagged seed investigations, which have been used in many previous studies [24], we conducted systematic investigations of caches in the ground. The fixed distance sample investigations reflected the overall dispersal situation of the Korean pine seeds outside the pine parent stands. In addition, the nest sampling investigations reflected the hoarding traits around nest trees as well as the space use of squirrels. Our results showed that the hoarding range of squirrels was much larger than the normal search range, which is consistent with reports by Xiao (2005) [24]. Therefore, tagged seed methods may not be suitable for studying hoarding patterns of a species with a large hoarding range.

### Effect of food availability and animal space use on seed mass

We predicted that squirrels would hoard heavier pine seeds farther away from pine seed-bearing trees. The fixed distance sampling investigations indicated that squirrels hoarded heavier seeds 60 m away from pine stands but hoarded the lightest seeds under the pine seed-bearing trees (FIG. 3). This result supported our prediction and corroborated the findings of previous studies [17], [24], [25], [26], [37]. We also found that the squirrels selected heavier seeds to hoard when food availability was low (FIG. 3). Many studies have shown that hoarders face a relative high cache loss rate when food availability is low [15], [16], [26]; hoarding larger seeds farther away can serve to reduce pilferage from main storages [27].

We discovered two conflicting results in our research. First of all, the main factor affecting the hoarding seed mass was not hoarding distance but food availability based on the nest sampling investigations. The heaviest seeds were hoarded around nest trees during different years at different sample plots (FIG. 4, and compared to FIG. 3). These results did not support prediction 1. However, when we used radio-tracking methods to follow squirrel hoarding behavior, we found that the squirrels often immediately buried some seeds under the parent pine trees and then carried cone cores with the remaining seeds farther away [29], [32]. This behavior resulted in caches extending toward nest trees with the highest cache density being produced around the nest trees, which represented the core area of the squirrels’ home ranges in winter [29], [32]. Heavier seeds have a higher energy value and can provide a greater net income in the winter. Squirrels reduce their hoarding costs by leaving lighter seeds outside of their main hoarding area or by eating them immediately [29], [32]. This information suggests that the squirrels selectively hoarded the heaviest pine seeds in the core areas of their home range, regardless of its location, and their selectivity was not a response to lower food availability. The interannual differences in seed mass in the samples are most likely caused by fluctuations in seed mass and not seed production (see FIG. 2). Because most nest trees are outside the pine parent stands [29], [32], the uniformity of heavier seed mass in the 60-m samples in the fixed distance sampling investigations supported this conclusion.

The other conflicting result involves the difference in seed masses hoarded in different years. The heavier seed in the nest sampling investigations appeared in seed mast years instead of poor seed years as in the fixed distance sampling investigations. In the nest sampling investigations, the majority of the samples were located in the squirrels’ core hoarding areas, and whereas the caches in core hoarding area could reflect squirrels’ hoarding selectivity. Based on our results, the squirrels probably had large core hoarding areas with a lower cache density in poor seed years (FIG. 6, Table 1 and 2). Therefore, we were more likely to obtain samples with heavier seeds in the fixed distance sampling investigations in poor seed years than in seed mast years. Hence, it might be the reason for the conflicting result that the fixed sampling investigations were conducted without thinking about trait of animal space use.

The two conflicting results indicated that animal space use is an important factor that should be included in studies of hoarded seed mass patterns.

Effect of food availability and animal space use on cache density patterns

In accordance with our predictions, the data from the fixed distance sampling investigations showed clear declines in cache density with hoarding distance and lower seed production both throughout different years and between different sample plots, which indicates differences in food production (FIG. 5). These results supported prediction 2. Reducing cache density with distance can help to minimize cache losses because pilfers will expend a greater effort to find the next cache after incidentally discovering a cache [9], [17], [20]. A number of studies have shown that pilferage rates are strongly density dependent during low seed availability [1], [16], [26]. Hence, hoarders increase their hoarding distance in response to reductions in food availability and to obtain a greater net energy income in periods of food scarcity [17], [25], [38].

There were some conflicting results from the nest sampling investigations. The cache density decreased with an increase in hoarding distance in 2007, 2009 and 2010, which supported predictions 2 and 3. In contract, there was no distance effect detected in 2008 and 2011, which supported the results of Stapanian and Smith (1978). The cache densities were similar regardless of hoarding distance. Because pine seed production in 2008 and 2011 was extremely high (FIG 1), there may have been a threshold of food availability during these years. If food availability is high enough to satiate all hoarder and pilferer populations in a certain area, then the pilferage rate can be described as equation 1. In this equation, the gross food requirements of hoarders and pilferers in the area are expressed as constants *C_h_* and *C_p_*, respectively, while *H_r_* represents the rest of the hoarded food that would not be recovered by hoarders or pilferers.



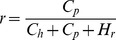
(1)


The pilferage rate, *r*, will decrease with the gross food hoarded based on equation 1. This equation indicates that an animal tends to increase its cache density and decrease hoarding distance to minimize cache losses and to gain more net energy when there is sufficient food available. Empirical studies have shown that animals do not cease hoarding behavior even during seed rich years [23]. When the food availability was above a certain threshold, such as in 2008 and 2011, all competitors were satisfied, and the squirrels made high and stable cache densities around nest trees (FIG 6).

In contrast to this satiation state, when food is scarce and the available food cannot satisfy the requirements of all the animals, the pilferage rate can be described by equation 2 or 3. The variables *R_h_* and *R_p_* represent the gross amount of hoarded food in a certain area that can be retrieved by hoarders and pilfers, respectively. The meaning of notation *H_r_* is the same as in equation 1. The *H_r_* will tend to zero in food scarcity situations.



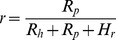
(2)




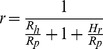
(3)


Because hoarders have a cache recovery advantage compared to pilfers [6], the ratio of *R_h_*/*R_p_* will increase with a lower cache density and decrease the pilferage rate *r*. Consequently, hoarders may create a lower density of caches to reduce the pilferage rate under conditions of food scarcity.

The idealized mathematical equations and our results indicated that a threshold of food availability might exist and that animals would employ different hoarding strategies in situations where food availability was beyond or below the threshold [14], [16]. However, determining the exact threshold is difficult because the factors and patterns that affect animal hoarding behavior are complex [17], [25].

Our results indicated that the degree of food availability and animal space use both determine animal hoarding patterns. The contradiction between Stapanian and Smith (1978) and Clarkson et al. (1986) may be due to the exclusion of the factors of animal space use and degree of food availability in those studies [1], [15].
